# The effect of magnetic field on the dynamics of gas bubbles in water electrolysis

**DOI:** 10.1038/s41598-021-87947-9

**Published:** 2021-04-30

**Authors:** Yan-Hom Li, Yen-Ju Chen

**Affiliations:** 1 Department of Mechanical and Aerospace Engineering, Chung-Cheng Institute of Technology, National Defense University, Taoyuan, 33551 Taiwan; 2 System Engineering and Technology Program, National Yang Ming Chiao Tung University, Hsin-Chu, 30010 Taiwan

**Keywords:** Electrocatalysis, Chemical hydrogen storage, Chemical physics, Fluid dynamics

## Abstract

This study determines the effect of the configuration of the magnetic field on the movement of gas bubbles that evolve from platinum electrodes. Oxygen and hydrogen bubbles respectively evolve from the surface of the anode and cathode and behave differently in the presence of a magnetic field due to their paramagnetic and diamagnetic characteristics. A magnetic field perpendicular to the surface of the horizontal electrode causes the bubbles to revolve. Oxygen and hydrogen bubbles revolve in opposite directions to create a swirling flow and spread the bubbles between the electrodes, which increases conductivity and the effectiveness of electrolysis. For vertical electrodes under the influence of a parallel magnetic field, a horizontal Lorentz force effectively detaches the bubbles and increases the conductivity and the effectiveness of electrolysis. However, if the layout of the electrodes and magnetic field results in upward or downward Lorentz forces that counter the buoyancy force, a sluggish flow in the duct inhibits the movement of the bubbles and decreases the conductivity and the charging performance. The results in this study determine the optimal layout for an electrode and a magnetic field to increase the conductivity and the effectiveness of water electrolysis, which is applicable to various fields including energy conversion, biotechnology, and magnetohydrodynamic thruster used in seawater.

## Introduction

Gas bubbles affect energy and mass transfer for electrode that produce gas. Bubbles that evolve at an electrode can block electrocatalyst surface^1,2^ and ion-conducting pathways in the electrolyte, resulting in energy losses^3^. Potential variation due to bubble generation depends on the operating current density, cell design (electrode geometry and interelectrode instance), and electrolyte composition. Several studies have estimated these losses under specific conditions^4–6^. Removing bubbles from the electrode and reducing energy losses during the electrolysis of water is worthy of study. Hydrogen is considered a potential energy carrier, because no greenhouse gases are produced. Among methods of hydrogen production, water electrolysis is the most common way because the produced hydrogen has high purity. Therefore, the topics for enhancement of the water electrolysis efficiency have drawn much attention over the past few decades. Recent studies report the effect of magnetic field on hydrogen production and its certain practical applications. Some studies use a magnetic field to induce a Lorentz force on the electrolyte^7,8^, which generates convection and increases mass transfer, and results in the decrease of internal resistance and concentration overpotentials. A convective flow also allows the desorption of bubbles and reduces their coverage of the electrodes^9–11^. A magnetic field perpendicular to the electrodes has been shown to reduce overpotentials^12^. The overpotential for hydrogen formation dropped by 10% when the electrodes were subjected to a magnetic field of 1.5 T. It was found that an external magnetic field has a strong influence on the electrolysis effectiveness. The property of the electrode material can also affect the efficiency of the electrolysis. Ferromagnetic materials give greater higher efficiency than paramagnetic materials or diamagnetic materials. The effectiveness of hydrogen production is significantly enhanced in a shorter inter-electrode under an upward Lorentz force^8^. Some studies show that the charging voltage decreases significantly when the distance of the electrode or the concentration of the solution is reduced. The voltage can be further decreased in the presence of a higher magnetic field because of the magnetohydrodynamic (denoted as MHD) effect^13,14^. Such MHD effect has been scaled up and widely employed to generate propulsion for a seawater vehicle or thruster^15,16^.

Many studies have reported that the behaviors of oxygen in water can be affected by an external magnetic field which would enhance the dissolving efficiency of the oxygen or the interface flow velocity^17–23^. Most of the results show that when a field strength less than 1 T is applied to the solution, the concentration of the dissolved oxygen is increased up to 50%. The paramagnetic property of oxygen molecules makes it possible to control the movement of the oxygen in the solution by an external magnetic field^24^. Oxygen bubbles will move directionally along the surface of the anode when a magnet is faced with the anode. Besides, oxygen bubbles would be in rotational motion in a square electrolyzer, which confines the directional locomotion of oxygen bubbles^25^. On the other hand, gas bubbles produced by the reduction and oxidation reaction can propel conducting objects or microswimmers that are applicable to biotechnology^26,27^.

According to the related research mentioned above, bubbles in electrochemical processes induce convection and increase mass transfer rates and an external magnetic field increase the effectiveness of electrolysis^28–32^. Numerous studies show the MHD effects on the polarized potential and effectiveness of the water electrolysis. Most of them demonstrate the potential or current density can be changed by the MHD effect (Lorentz force). Fewer reports have showed the locomotion mechanism of the bubbles and how may a configuration of magnetic field affect the movement of the bubbles and the effectiveness of the water electrolysis. The relationship between bubbles movement and the magnetic field is not thoroughly understood. Increasing the efficiency of electrolysis processes requires a detailed understanding of the behavior of bubbles in electrochemical systems that are subjected to a magnetic field.

This study uses platinum material as the electrode because it features high electrical conductivity, is resistant to corrosion, and exhibits paramagnetic characteristics. Various configurations of platinum electrodes and magnetic field are used to determine the effect of a parallel and perpendicular field on the movements of the hydrogen and oxygen bubbles during the evolution reaction. The mechanism by which bubbles move under the influence of a magnetic field are investigated. The conductivity between the electrodes is measured to determine the optimal layouts for electrodes and magnetic field that increases the charging performance and the effectiveness of electrolysis. The results in this study are applicable to various fields including energy conversion, biotechnology^26,27^, and an MHD thruster used in seawater^15,16^.

## Materials and method

### Theory

During the electrolysis process, the water molecules are dissociated into hydrogen (H_2_) and oxygen (O_2_) under the influence of electricity. There are four types of water electrolysis based on the electrolyte, operating conditions, and ionic agents (OH^−^, H^+^, O^2−^), however, operating principles and the overall reactions are the same. The four types of electrolysis methods are (1) alkaline water electrolysis (AWE)^33–35^, (2) solid oxide electrolysis (SOE)^36,37^, (3) microbial electrolysis cells (MEC)^38,39^, and (4) PEM water electrolysis^40,41^. For pure water electrolysis, the acid-balanced reactions that occur at the anode and cathode surfaces are:

H^+^ reduction at the cathode:

1 $$2{\text{H}}^{ + } \left( {aq} \right) + 2{\text{e}}^{ - } \to {\text{H}}_{2}$$

H_2_O oxidation at the anode:

2 $$2{\text{H}}_{2} {\text{O}}(l) \to {\text{O}}_{2} \left( g \right) + 4{\text{H}}^{ + } \left( {aq} \right) + 4{\text{e}}^{ - }$$

The overall reaction is as follows:

3 $$2{\text{H}}_{2} {\text{O}} \to 2{\text{H}}_{2} + {\text{O}}_{2}$$

During water electrolysis, gas production is proportional to the electric current. This study plots the I–V curves and compare the conductivity of the electrolyte, with and without a magnetic field. The difference in conductivity (denoted as ΔG) and the increase rate of conductivity (*η*_G_) are thus defined as:

4 $$\Delta {\text{G}} = {\text{G}}_{{{\text{magnetic}}}} - {\text{G}}_{{\text{no magnetic}}}$$

5 $$\eta_{{\text{G}}} = \left( {{{\Delta {\text{G}}} \mathord{\left/ {\vphantom {{\Delta {\text{G}}} {{\text{G}}_{{\text{no magnetic}}} }}} \right. \kern-\nulldelimiterspace} {{\text{G}}_{{\text{no magnetic}}} }}} \right) \times 100\%$$

where the G_magnetic_ and G_no magnetic_ are the respective conductivity between the electrodes with and without an external field.

### Material preparation

The electrode material is platinum with an area of 50 mm × 50 mm. Platinum is a transition metal that exhibits paramagnetism and high chemical stability, so it is helpful to enhance the movement of the gas bubbles in a magnetic field. The electrolyte is the distilled water without mixing any acid or alkali solution at room temperature. The experiments were conducted in a water tank with dimensions of 400 mm × 240 mm × 200 mm. Two pieces of N35 NdFeB magnet with a surface magnetic field strength of 0.22 T and with dimensions of 100 mm × 50 mm × 10 mm were used to generate a magnetic field parallel or perpendicular to the electrodes. A power supply (GWInstek APS-1102) with a maximum current of 10 A was used to supply a fixed current and the charging voltage for the various experimental layouts was measured.

### Experimental layout

**Figure 1:**
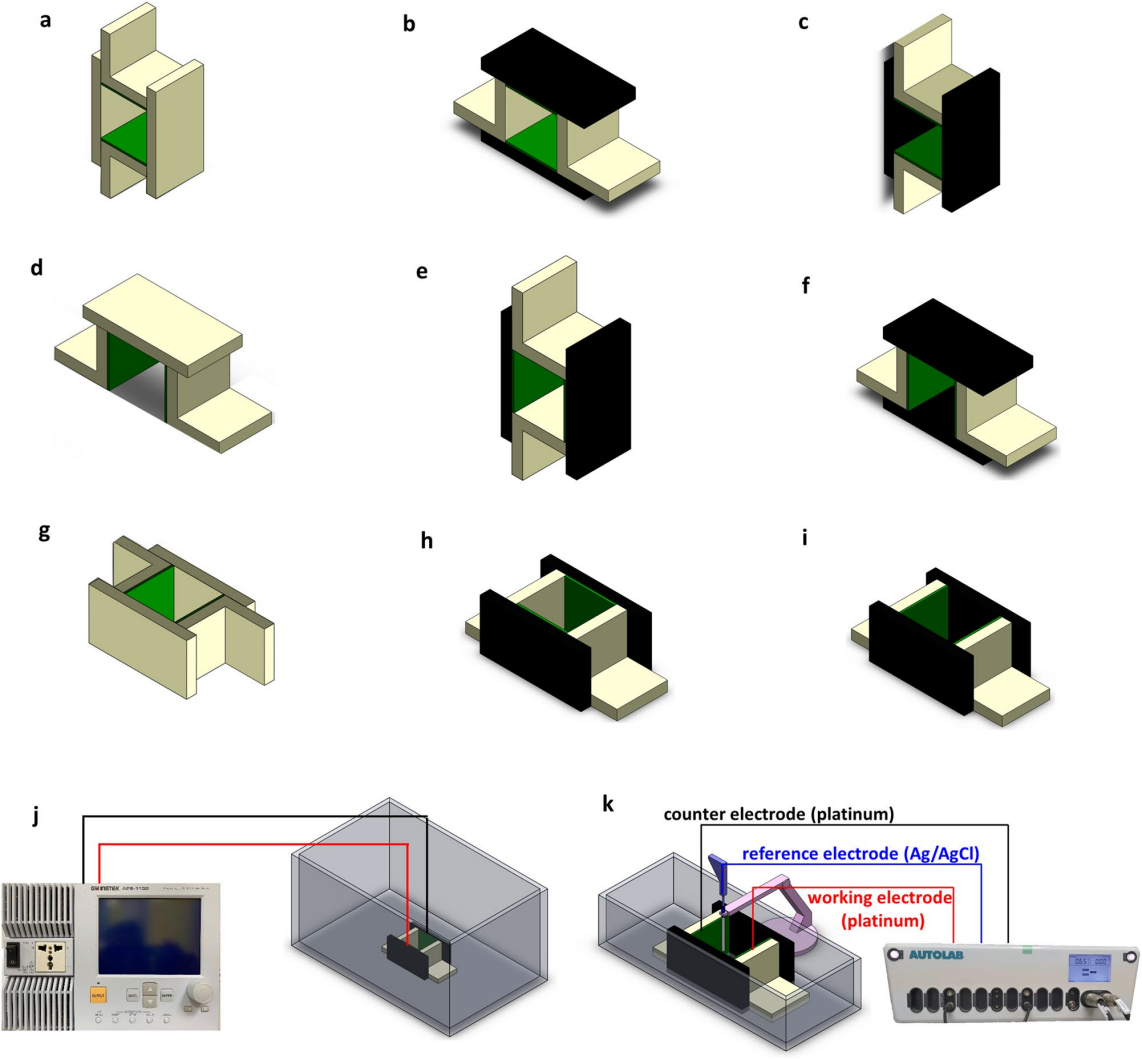
A schematic diagram of the experimental setup and the layout of the electrodes and the magnetic field. The black area is a pair of N35 magnets (100 mm long, 50 mm wide, and 10 mm high) whose surface field strength is nearly 0.22 T, the green part is the electrodes (50 mm long and 50 mm wide) and the yellow part is the isolating plastic that is used to form the duct. Various magnetic field configurations were used to determine the effect of the field on the behaviors of the gas bubbles: (**a**) electrodes placed horizontally without a magnetic field. (**b**) Electrodes placed horizontally with two horizontal magnets. (**c**) Electrodes placed horizontally and perpendicular to the magnets to create a Lorentz force (denoted as F_L_) in the horizontal direction. (**d**) Electrodes placed vertically without a magnetic field. (**e**) Electrodes placed vertically with vertical magnets. (**f**) Electrodes placed vertically and perpendicular to magnets to create a Lorentz force in the horizontal direction. (**g**) Electrodes placed vertically without a magnetic field and with an upward exit. (**h**) Electrodes placed vertically with vertical magnets and an upward exit. (**i**) Electrodes placed vertically and perpendicular to the magnets to create a Lorentz force in the vertical direction and with an upward exit. (**j**) All the experiments were conducted in a 40 cm long, 24 cm wide, and 20 cm high water tank. The electrodes were connected to a power supply (GWInstek APS-1102) that generates a maximum current of 10 A. (**k**) Schematic diagram of a three-electrode cell for the potentiometry measurements.

The effect of various magnetic field configurations on the behaviors of the gas bubbles were investigated experimentally, as shown in Fig. 1. Figure 1a–c show the layouts for the horizontal electrodes with no magnetic field and a magnetic field perpendicular and parallel to the electrodes. Figure 1d–f show the layouts for the vertical electrodes with no magnetic field and a magnetic field parallel and perpendicular to the electrodes. Figure 1g–i show the layouts for the vertical electrodes with an upward outlet and no magnetic field and a magnetic field parallel and perpendicular to the electrodes to determine the effect of a vertical force on the conductivity of the system. All the experiments were conducted in a 40 cm long, 24 cm wide, and 20 cm high water tank, as shown in Fig. 1j. The electrodes were connected to a power supply (GWInstek APS-1102) that generates a maximum current of 10 A. To further investigate action mechanism of magnetic field on gas bubbles movement, electrochemical measurements were carried out using a potentiostat (AutoLab PGSTAT302N) in a three-electrode cell configuration, as shown in Fig. 1k. All the electrochemical measurements were carried out at room temperature. Potentiometry scan curves were measured for the current density of 200 A/cm^2^ at constant scan rate of 10 mV/s. The platinum sheets were used as a working electrode (WE) and a counter electrode (CE). A gel-type electrolyte Ag/AgCl electrode filled with 3 M KCl served as the reference electrode was immerged at the center of the cell.

## Results

### The effect of a perpendicular magnetic field

To determine the effect of a magnetic field (denoted as B) that is vertical to the surface of the electrode, the magnets are placed parallel to the electrodes, as the layouts shown in Fig. 1b,e,h. This perpendicular magnetic field results in a gradient magnetic force (denoted as F_▽B_), which is caused by the electrogeneration of paramagnetic molecules in a non-uniform magnetic field and affects the behaviors of the gas bubbles evolved from a paramagnetic platinum electrode^8,31^. The equation for calculating F_▽B_ is defined as follows^42,43^:

6 $${\text{F}}_{{{\triangledown }{\text{B}}}} = {{\upchi}}_{{\text{m}}} {\text{cB}}{\triangledown }{\text{B}}/{{\upmu}}_{0}$$

where χ_m_ is the molar magnetic susceptibility, c is the concentration, B is the magnetic flux density, ▽B is the magnetic flux density gradient and μ_0_ is the magnetic permeability of free space.

### The effect of a parallel magnetic field (MHD effect)

The effect of a magnetic field that is parallel to the surface of the electrode is different to that of a magnetic field perpendicular to the electrode. The layouts for a magnetic field that is parallel to the electrode are shown in Fig. 1c,f,i. However, it is difficult to magnetize an electrode if the magnetic field is parallel to the surface of the electrode. The mechanism for the movement of the gas bubbles that evolved from the electrodes is the Lorentz force (denoted as F_L_), which is a force exerted on a charged particle moving in an electricmagnetic field. Only a suitable magnetic field direction generates a Lorentz force and enhances the convective phenomenon for water electrolysis. The magnitude of the Lorentz force that acts on the fluid in the duct of MHD system is given as^15^:

7 $${\text{F}}_{{\text{L}}} = wB_{{{\text{eff}}}} I$$

where F_L_ is the Lorentz force, *B*_eff_ is the effective field, which depends on the design parameter of the MHD configuration, *I* is the current, and *w* is the distance between the electrodes. A magnetic field is added to the electric field to increase the rate of electrolysis because charged particles are forced in a direction that is perpendicular to the magnetic lines as the magnetic equivalent lines and electrical equivalent lines are orthogonal. An optimal layout for the magnetic and electric directions will yield a uniform Lorentz-induced flow and increase the rate of electrolysis.

If a magnetic field is uniform, F_▽B_ can be neglected because its value is much less than F_L_ if a parallel magnetic field is applied^14^. Therefore, the effect of a magnetic field on the behavior of gas bubbles depends mainly on F_L_. However, for a non-uniform magnetic field environment, the perpendicular magnetic field F_L_ is much less than F_▽B_^42,44^. For different scenarios, the effect of a magnetic field on the behaviors may be mainly due to F_L_ or F_▽B_.

### The effect of the configuration of a magnetic field on the gas bubbles that evolve from horizontal electrodes

**Figure 2:**
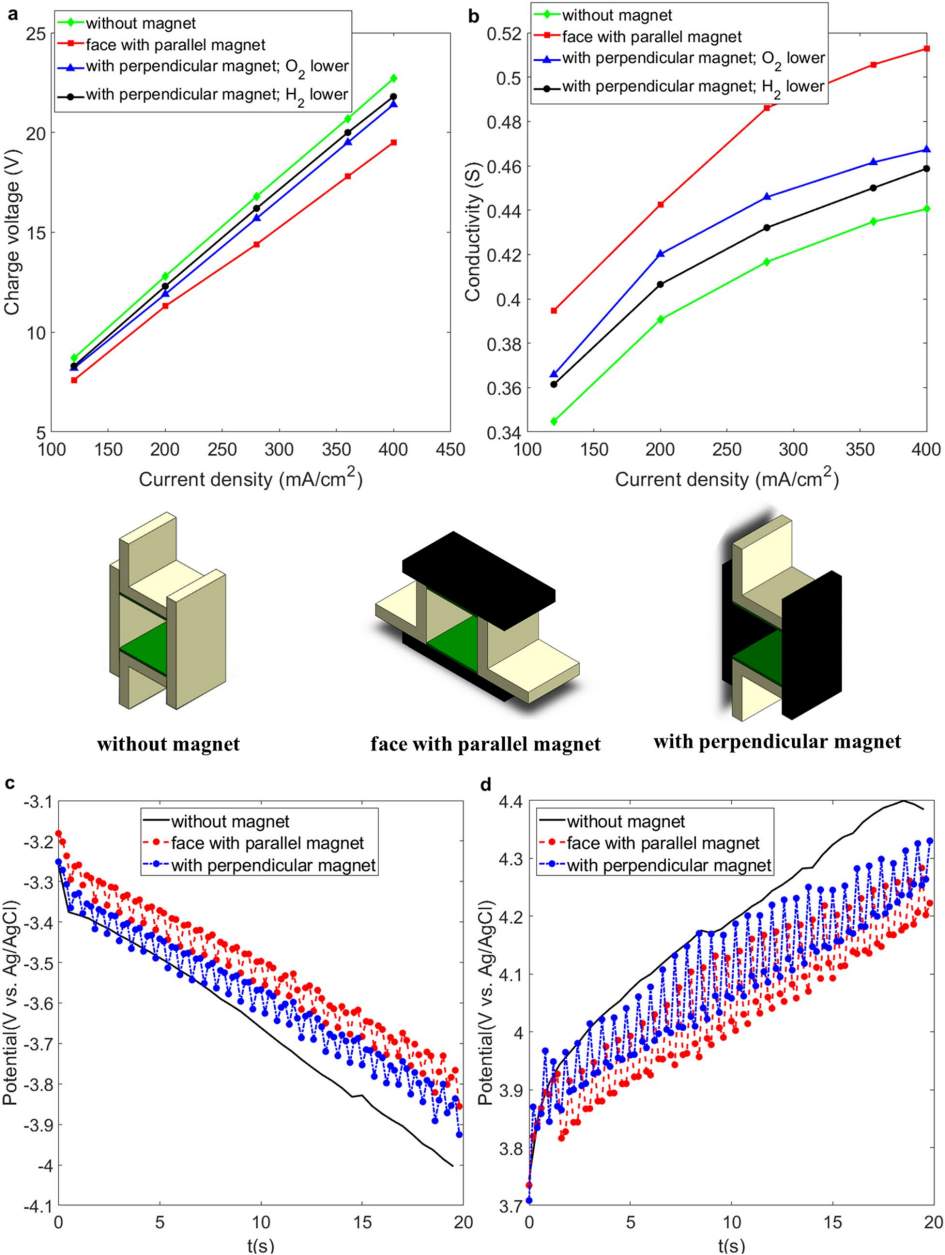
Comparison of the conductivity for horizontal platinum electrodes in various configurations of magnetic field. (**a**) Charging voltage vs. current density for the experimental layouts shown in Fig. 1a–c. (**b**) Conductivity vs. current density for the experimental layouts shown in Fig. 1a–c. (**c**) Polarized potential curve for the reduction reaction (cathode). (**d**) Polarized potential curve for the oxidation reaction (anode). The potential was measured at current densities of 200 mA/cm^2^ with the scan rate of 10 mV/s.

Figure 2a shows that the charging voltage increases linearly as the current density increase in the platinum electrodes that are placed horizontally under various field layouts, as shown in Fig. 1a–c. Figure 2b shows the effect of a magnetic field on the conductivity between the two platinum electrodes. The applied magnetic field reduces the charging voltage if the electrodes are faced with the magnets or perpendicular to the magnets. This result shows both a parallel and a perpendicular magnetic field increase the conductivity between the electrodes. A perpendicular magnetic field that is generated by the parallel magnets generates a greater increase in the conductivity of horizontal electrodes.

Figure 2a shows that for the same current density of 200 mA/cm^2^, generated by an electrical current of 5 A, horizontal electrodes with parallel magnets have the lowest charging voltage of 11.3 V, which correlates to the highest conductivity of G = 0.443, as shown in Fig. 2b. The increasing ratio *η*_G_ is about 13.3% greater than the ratio without a magnetic field. The effects of magnetic field on the potentiometry curve for the layout in Fig. 1b,c are shown in Fig. 2c,d, which respectively demonstrate the distribution of the polarized potential for the reduction (cathode) and oxidation (anode) process in 20 s. The polarized potentials increase over time due to the bubbles that constantly evolve from the surfaces of the anode and cathode. The potential decreases and the oscillation occurs because the bubbles periodically evolve and detach from the electrodes when the magnetic field is applied. Figure 2c,d show that the horizontal electrode in the perpendicular magnetic (parallel magnet) field has more significant decrease in polarized potential than that in parallel magnetic field (perpendicular magnet). This trend corresponds to the result of the comparison of the conductivity shown in Fig. 2b.

**Figure 3:**
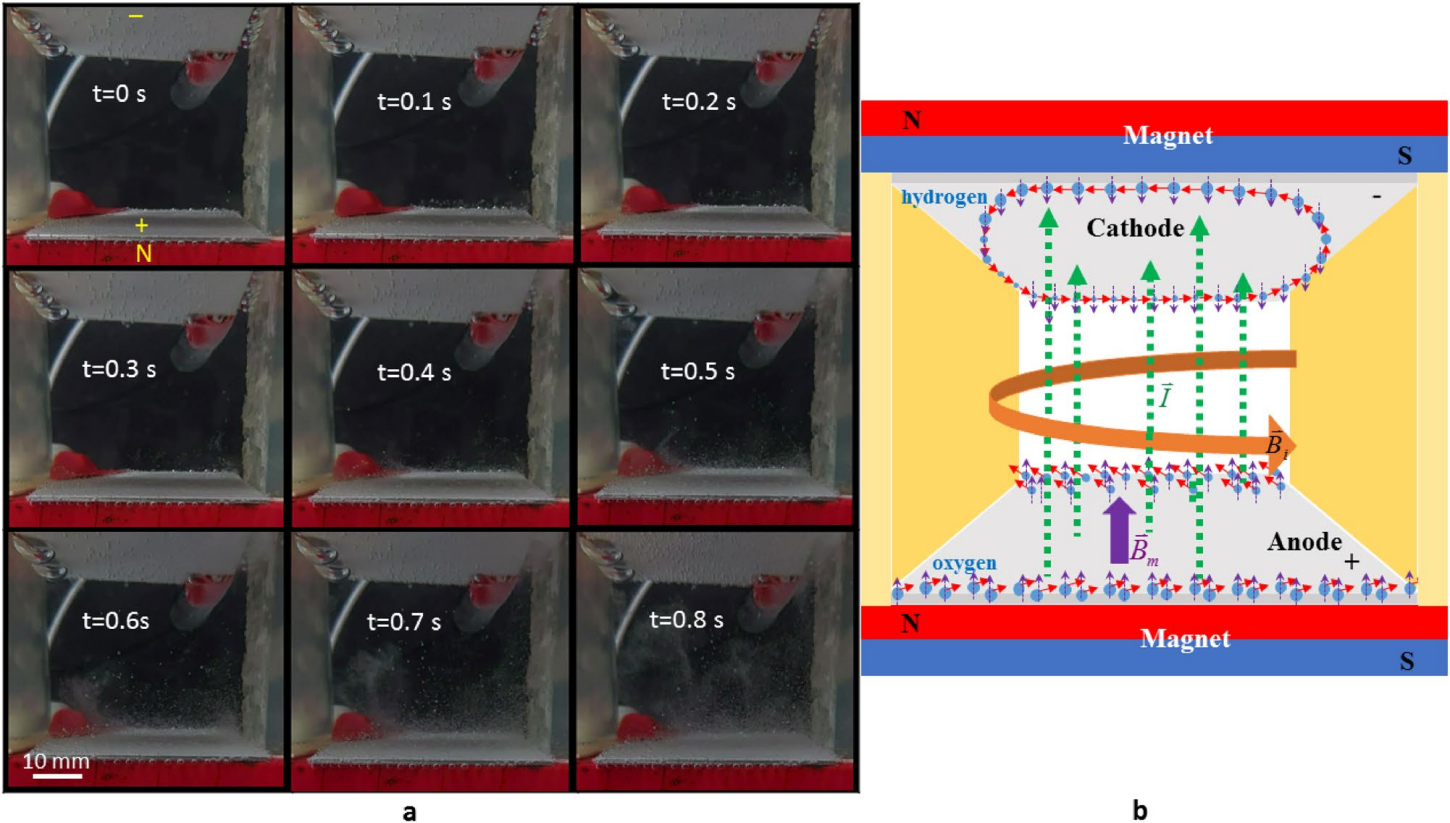
Swirling gas bubbles in a magnetic field that is perpendicular to the electrode surface. (**a**) Sequential images of the locomotion of the oxygen (lower electrode) and hydrogen (upper electrode) on the platinum electrodes in a perpendicular magnetic field. The experimental layout is shown in Fig. 1b with an electrical current of 5 A. (**b**) Schematic diagram of the revolution mechanism for the oxygen and hydrogen bubbles. The green-dash arrows are the current $${\overset{\lower0.5em\hbox{$\smash{\scriptscriptstyle\rightharpoonup}$}} {I} }$$ produced by the charging particles and ions flowing in the duct. $$\overset{\lower0.5em\hbox{$\smash{\scriptscriptstyle\rightharpoonup}$}}{B} _{i}$$ is the cylindrical magnetic field created by the current $${\overset{\lower0.5em\hbox{$\smash{\scriptscriptstyle\rightharpoonup}$}} {I} }$$. The purple-dash arrows represent the induced magnetic field of the bubbles caused by the applied magnetic field $$\overset{\lower0.5em\hbox{$\smash{\scriptscriptstyle\rightharpoonup}$}}{B} _{m}$$. The red arrows are the moving direction of the bubble.

Figure 3a shows the movement of the gas bubbles in a duct between two horizontal electrodes in a perpendicular magnetic field. The circular movements are found for both the oxygen and hydrogen bubbles (see Supplementary video 1). The paramagnetic oxygen bubbles spread away from the anode surface (lower electrodes) and make a counterclockwise revolution when the N-pole of the magnet faces with the anode, then the upward buoyancy force results in a swirling upward motion for the oxygen bubbles (see Supplementary video 2). Figure 3b shows the proposal explanation for the mechanism of the bubble movement in the perpendicular magnetic field. The oxygen bubbles that evolve from the anode are magnetized by the external magnetic field and have induced magnetic fields in the direction of the applied magnetic field because of their paramagnetic nature. When the charging particles or ions flow in the duct between the electrodes, the duct can be regards as a straight wire, that creates a circular or cylindrical magnetic field around the duct according to the right-hand rule. The cylindrical magnetic field induces the paramagnetic oxygen bubbles to make a counterclockwise revolution when the N-pole of the magnet faces with the anode.

On other hand, the hydrogen bubbles evolving from the cathode (upper electrode) that faces S-pole of the magnet almost adhere to the cathode surface because of the buoyancy force and rotate slowly in a clockwise direction, as viewed from the top. Hydrogen bubbles revolve in the opposite direction to oxygen bubbles because of the diamagnetic nature of hydrogen molecular. Additionally, oxygen and hydrogen bubbles revolve in reverse direction when the poles of the magnets that face the anode and cathode electrode are changed (see Supplementary video 3). The difference in the direction in which the upper and lower bubbles rotate results in a twisted flow between the electrodes, which serves as a stirrer to increase hydrodynamics. This phenomenon shows that the horizontal rotation due to the interaction between the paramagnetic/diamagnetic bubbles and the magnetic field allows oxygen and hydrogen bubbles to detach from the surface of the electrodes and reduces the internal resistance.

**Figure 4:**
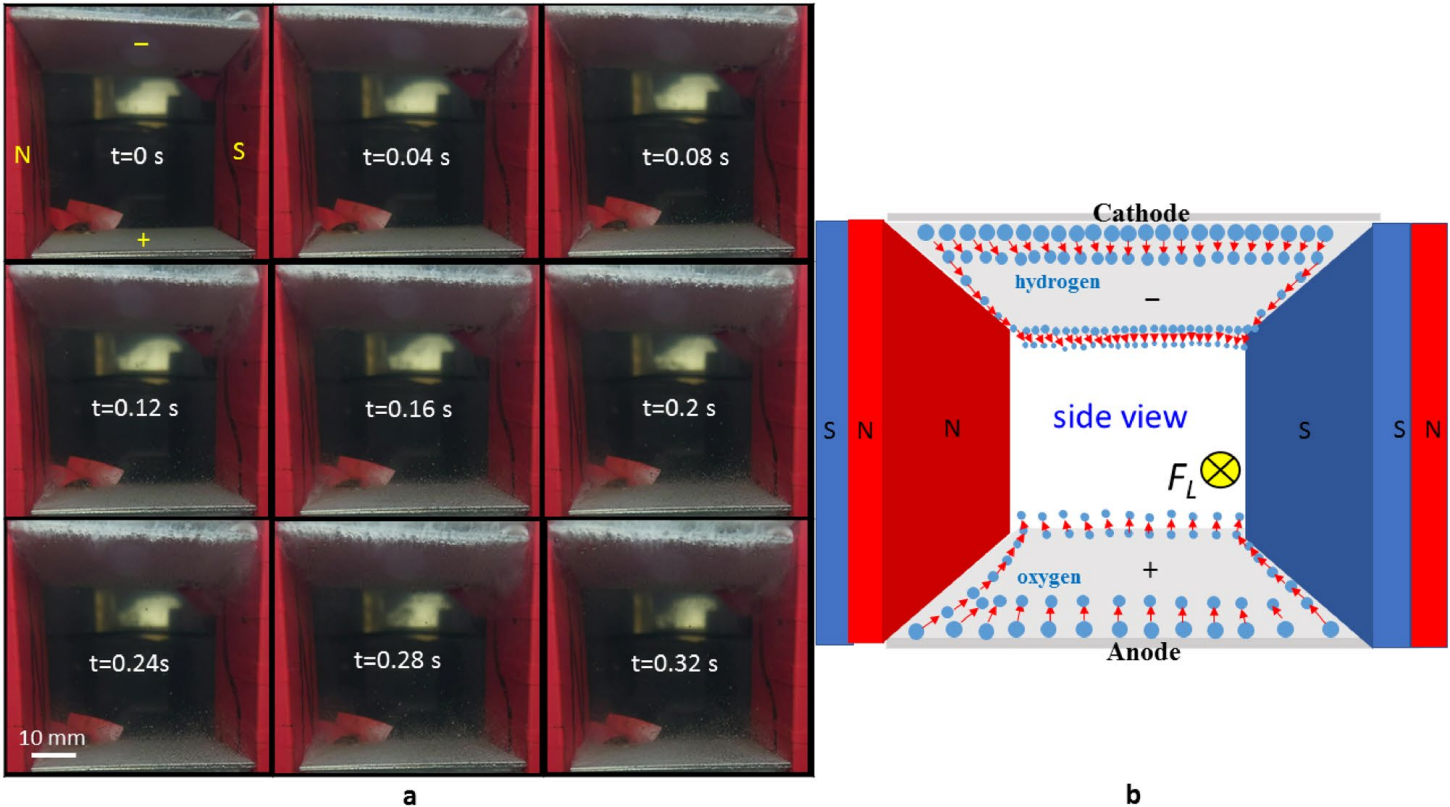
Locomotion of gas bubbles that are produced by horizontal platinum electrodes under the effect of a horizontal Lorentz force that is generated by a horizontal magnetic field and a perpendicular electric field. (**a**) Sequential images of the oxygen and hydrogen bubbles that are detached from the lower and upper platinum electrode, respectively. The experimental layout is shown in Fig. 1c for the electrical current of 5 A. The N-pole and S-pole of the magnet are at the left and right sides, respectively. (**b**) Schematic diagram of the locomotion of the gas bubbles.

For horizontal electrodes in a parallel magnetic field that is produced by the vertical magnets, the movement of the gas bubbles is different to that in Fig. 3. The locomotion of the gas bubbles is mainly caused by the horizontal Lorentz force (MHD effect) that is created by orthogonal electrical and magnetic fields. For the field configuration shown in Fig. 4, the direction of Lorentz force is into the diagram. Oxygen bubbles detach from the surface of the anode when the concentration of oxygen bubbles near the surface reaches saturation (see Supplementary video 4). The Lorentz-force-induced flow rapidly flushes oxygen bubbles in an inward direction. Thus, the oxygen bubbles do not adhere to the surface of the anode or stay in the duct for a long time and the internal resistance and charging voltage for this MHD system decrease. Equation (3) states that the number of hydrogen bubbles that evolve from the cathode is twice the number of the oxygen molecular that evolve from the anode and these mostly gather on the cathode surface due to the upward buoyancy force. This inhibits the movement of the hydrogen bubbles and decreases the overall conductivity of the fluid in the duct. This explains the lower conductivity of G = 0.42 compared to G = 0.443 of the parallel magnets as shown in Fig. 2b for an identical current density of 200 mA/cm^2^.

**Figure 5:**
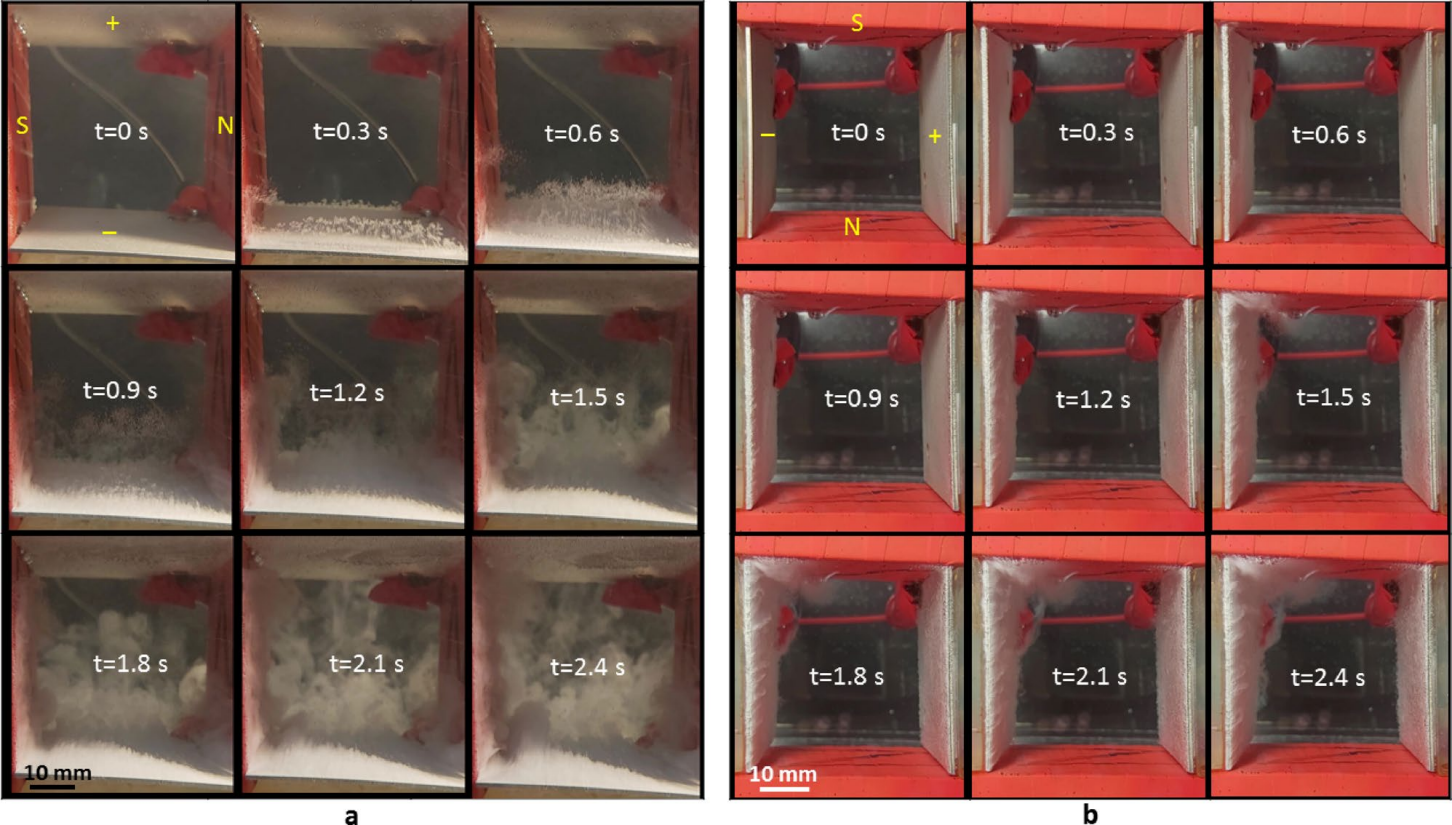
Locomotion of gas bubbles under the effect of a horizontal Lorentz force using a different experimental layout. (**a**) Sequential images of the oxygen and hydrogen bubbles that are detached from the upper and lower platinum electrode, respectively. The experimental layout is shown in Fig. 1c. The N-pole and S-pole of the magnet are at the right and left sides, respectively, for the electrical current of 5 A. (**b**) Sequential images of oxygen and hydrogen bubbles that are detached from the right and left platinum electrodes, respectively. The experimental layout is shown in Fig. 1f. The N-pole and S-pole of the magnet are at the lower and upper sides, respectively.

If the hydrogen bubbles evolve from the lower electrode, as shown in Fig. 5a, the distribution pattern is significantly different to that in Fig. 4a. Figure 5a shows that the gas bubbles in the dust are denser than that shown in Fig. 4a and almost scattered in the whole dust within a show period (see Supplementary video 5). This is because the hydrogen bubbles are lighter and more hydrogen bubbles evolve from the cathode electrode than oxygen bubbles form the anode. When the concentration of hydrogen bubbles near the surface reaches saturation, hydrogen bubbles rapidly spread away from the surface of the cathode due to the effect of the upward buoyancy force. However, the inward Lorentz force is not sufficiently strong to spread the bubbles in the dust toward the exit. As a result, the internal resistance of the electrode-magnet layout shown in Fig. 5a is greater than that for Fig. 4a. The conductivity of G = 0.406 for the cathode at the lower position is less than G = 0.42 for the anode at the lower position for an identical current density of 200 mA/cm^2^.

### The effect of the configuration of a magnetic field on gas bubbles that evolve from vertical electrodes

**Figure 6:**
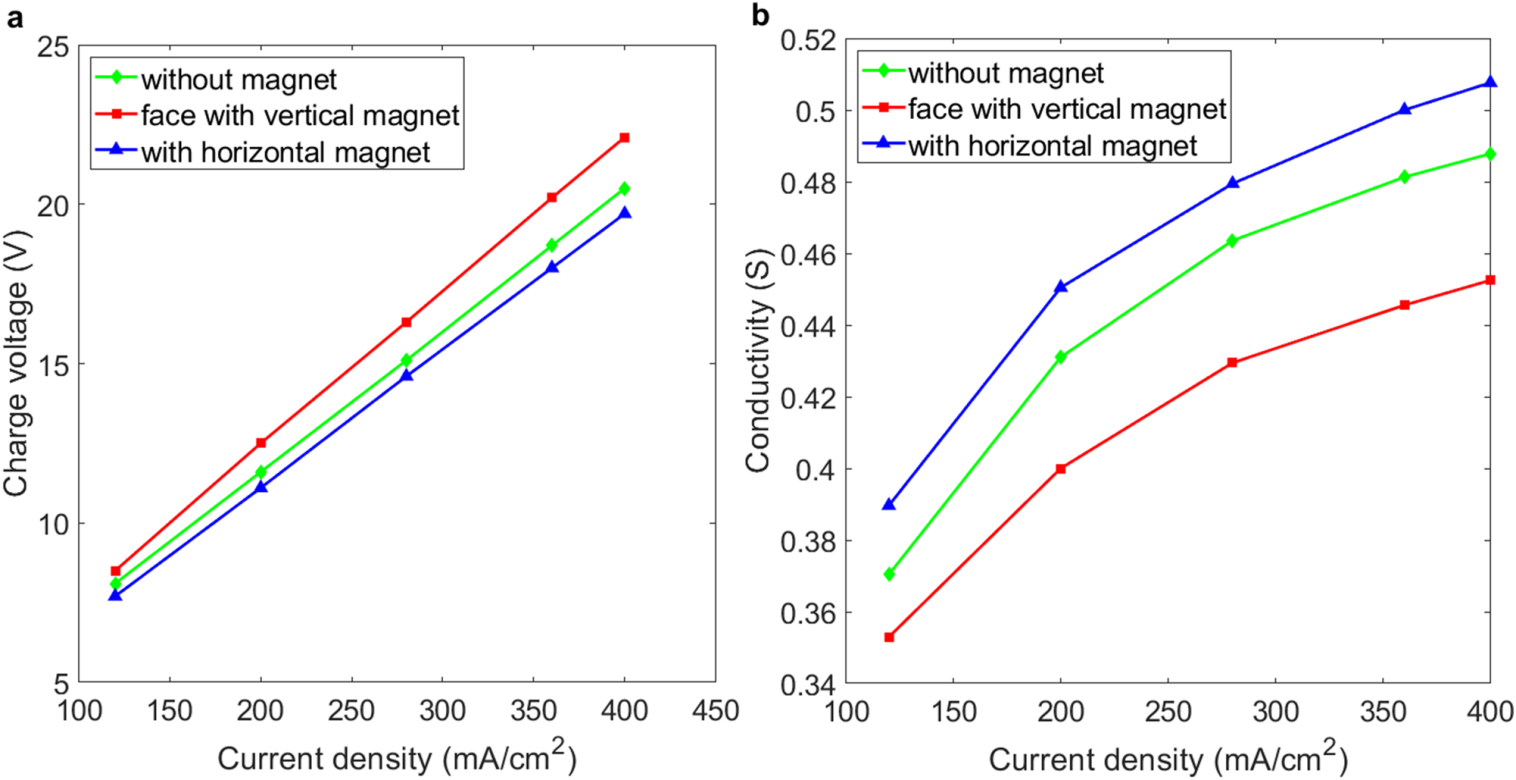
Comparison of the conductivity for vertical platinum electrodes in various magnetic field configurations. (**a**) Charging voltage vs. current density for the experimental layout in Fig. 1d–f. (**b**) Conductivity vs. current density for the experimental layouts in Fig. 1d–f.

To eliminate the effect of the buoyancy force on the stagnation of the bubbles on the upper electrode, experiments involving vertical electrodes in the presence of various magnetic field configurations that are shown in Fig. 1d–f are performed. Figure 5b shows the movement of the gas bubbles for the layout in Fig. 1f, which are turned 90° clockwise from Fig. 5a. There are significantly fewer gas bubbles in the duct, compared to Fig. 5a. When the respective concentrations of oxygen and hydrogen bubbles near the anode (right) and the cathode (left) surface reaches saturation, the inward Lorentz force rapidly flushes the bubbles out of the exit (see Supplementary video 6). The effect of buoyancy force is seen from t = 1.5 s to t = 2.4 s near the corner between the cathode and the magnet S-pole when the number of the hydrogen bubbles is increased gradually. For the layout in Fig. 5b, the horizontal Lorentz force has a greater effect than the upward buoyancy force. Thus, significantly fewer bubbles stay in the dust and conductivity is increased, as shown in Fig. 6.

Figure 6a shows that the charging voltage increases linearly as current density increases in vertical platinum electrodes placed in various field conditions, as shown in Fig. 1d–f. For this electrode-magnet layout at the same current density of 200 mA/cm^2^, which is generated by an electrical current of 5 A, the vertical electrodes with parallel magnets shows the lowest charging voltage of 11.1 V, which corresponds to a conductivity of G = 0.45 in Fig. 6b, and there is a 4.7% increase in the ratio *η*_G_, compared to no magnetic field. This is better than the respective conductivity values of G = 0.42 and G = 0.40 for the layouts shown in Figs. 4a and 5a.

If vertical electrodes face vertical magnets, the charging voltage is increased from 11.1 to 12.5 V and the conductivity decreases from 0.45 to 0.4, which is less than the value of G = 0.431 for electrodes with no magnetic field, for an electrical current of 5 A. This result shows that the vertical rotation of bubbles due to the interaction between the paramagnetic and diamagnetic bubbles and the magnetic field does not allow oxygen and hydrogen bubbles to detach from the electrodes surface efficiently.

### The effect of a vertical Lorentz force on the movement of bubbles

**Figure 7:**
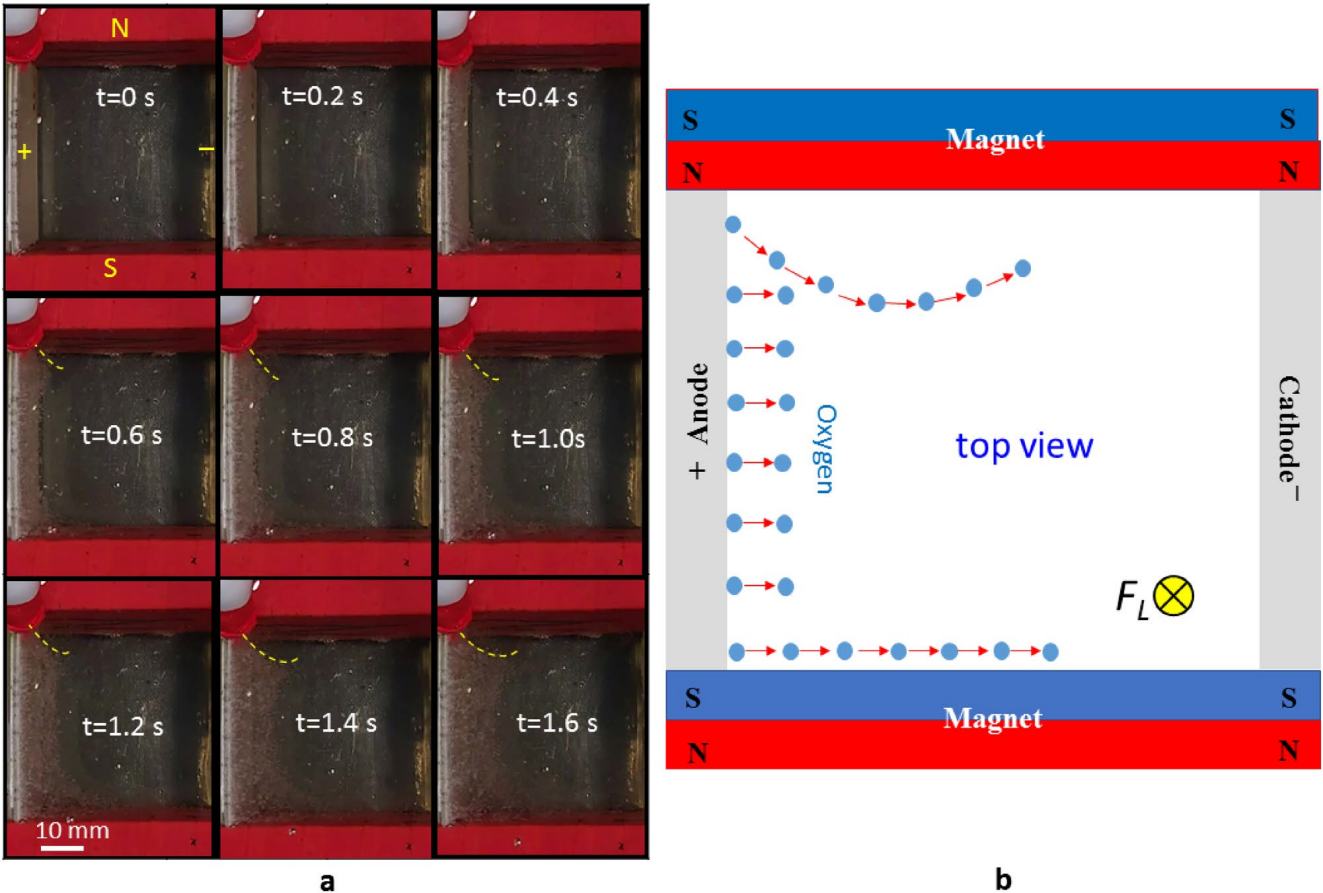
Repulsion of gas bubbles in a magnetic field that is parallel to the surface of the electrode. (**a**) Sequential images of oxygen bubbles that are detached from the left platinum electrode and respectively repelled and attracted by the N-pole and S-pole of the magnet. The experimental configuration is shown in Fig. 1d, for an electrical current of 5 A. (**b**) Schematic diagram of the locomotion and repulsion of the oxygen bubbles.

The experiments for the layouts shown in Fig. 1g–i determine the effect of a vertical Lorentz force and the vertical revolution of the bubbles on the conductivity of the fluid in the duct. Figure 7 shows the movement of the bubbles for vertical electrodes perpendicular to vertical magnets, which produces a vertical Lorentz force. The sequential images in Fig. 7 show that the oxygen bubbles move rightward and perpendicular to the surface of the anode when the concentration of oxygen bubbles near the anode surface reaches saturation. The cluster of oxygen bubbles near the N-pole of the magnet are repelled from the surface of the magnet and are attracted to the S-pole of the magnet (see Supplementary video 7). This result is caused by the paramagnetic nature of oxygen bubbles, which are magnetized by the external magnetic field and are weakly repelled by the magnet N-pole and attracted by the S-pole, as shown in the schematic diagram in Fig. 7b. The results in Fig. 7a also show the effect of the downward Lorentz force (F_L_) on the movement of the bubbles. Numerous studies show that the F_L_ that is produced by the charges moving under an applied magnetic field induces convection in the electrolyte^45–49^. Electrochemical reactions are improved by an increase in mass transport, which is enhanced by F_L_^45–52^. However, the direction in which F_L_ acts determines the efficiency of the reaction. A downward F_L_ competes with the upward buoyancy force, so the oxygen bubbles spread away from the anode surface more slowly. It is seen that the oxygen bubbles that are repelled by the N-pole magnet make a curving path and increases the moving distance. As a result, the oxygen bubbles stay longer in the duct of a layout that is subject to a downward Lorentz force. This type of layout results in a greater internal resistance and requires a higher charging voltage than the vertical electrodes in no magnetic field, as shown in Fig. 1g.

**Figure 8:**
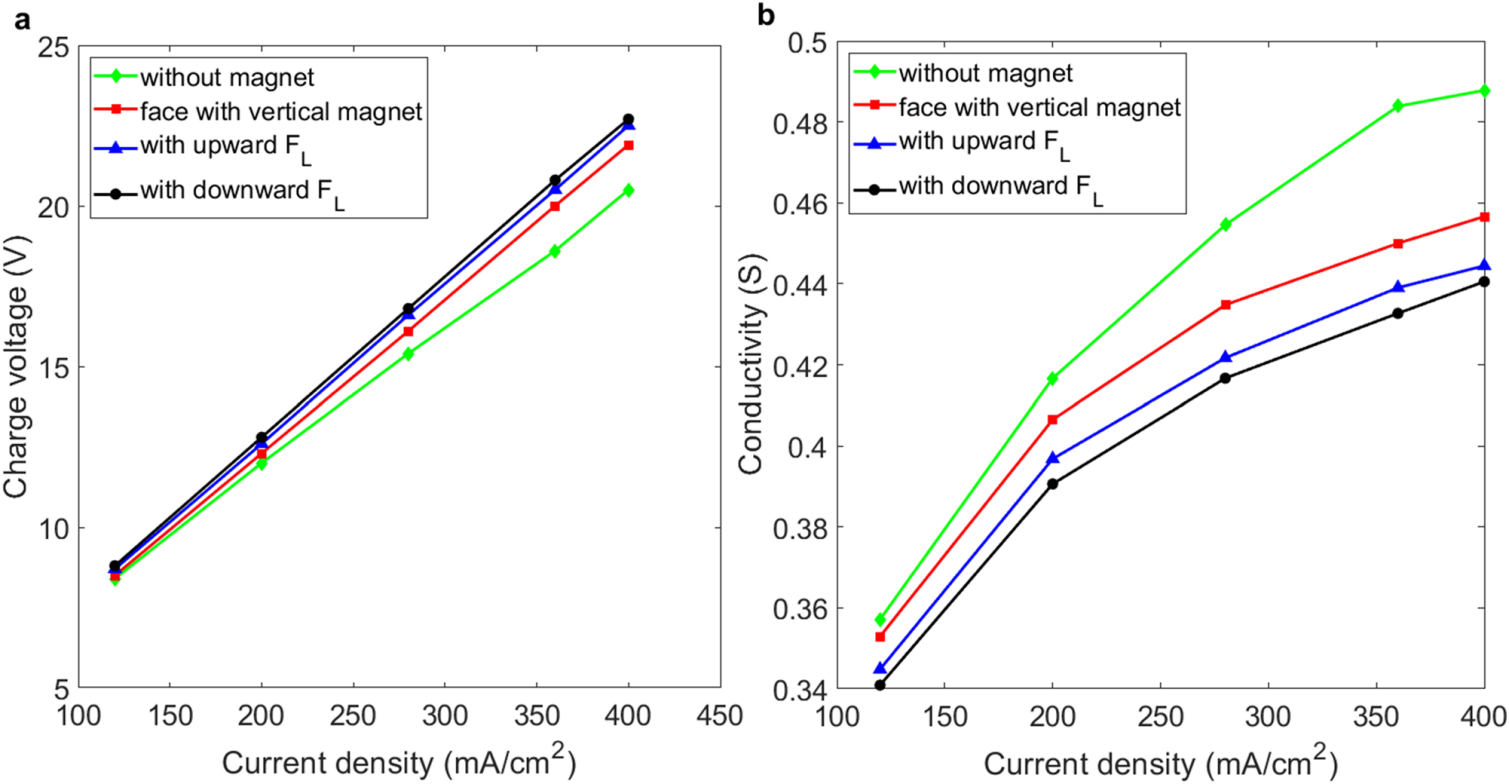
Comparison of the conductivity for vertical platinum electrodes in various configurations of magnetic field which constitute a duct with an upward exit. (**a**) Charging voltage vs. current density for the experimental layout in Fig. 1d–f. (**b**) Conductivity vs. current density for the experimental layout in Fig. 1d–f.

Figure 8a quantitatively compares the results for layouts with an upward exit in various field configurations. Figure 8b shows that an external magnetic field has a negative impact on the conductivity between the electrodes for the layout in Fig. 1h. At the same current density of 200 mA/cm^2^, which is generated by an electrical current of 5 A, vertical electrodes that are subject to a downward Lorentz force require a higher charging voltage of 12.8 V, which corresponds to a lower conductivity of G = 0.39, as shown in Fig. 8b. The increasing ratio *η*_G_ is about − 6.25% less than the ratio for no magnetic field.

The conductivity slightly increases when the bubbles are subject to an upward Lorentz force. An upward Lorentz force overcomes the weight of the static fluid in the duct and generates a Lorentz-force-induced flow. However, the closed bottom of the duct prevents sufficient inlet flow to continually generate a significant Lorentz force. Besides, the repulsion of the oxygen bubbles by the N-pole of the magnet increases the moving path of the bubbles and prolongs the period that they remain in the duct. As a result, an upward Lorentz force does not reduce the charging voltage for a layout shown in Fig. 7. The conductivity is merely increased from G = 0.39 to 0.396, which is less than the value of G = 0.417 for the layout with on magnetic field.

If vertical magnets face vertical electrodes for an upward-exit layout, the charging voltage is also greater than the electrodes that in no magnetic field. As shown in Fig. 8a, for vertical electrodes facing vertical magnets, the charging voltage is increased from 12 to 12.3 V, and the conductivity decreases from G = 0.417 to G = 0.407.

These results show that neither a vertical revolution for bubbles nor a vertical Lorentz reduce the internal resistance for a duct with an upward-exit layout, which means the external magnetic field does not increase conductivity for the configuration in Fig. 1g.

## Discussion

A magnetic field that is perpendicular to the surface of an electrode causes bubbles to revolve. For horizontal electrodes, oxygen and hydrogen bubbles that respectively evolve from the anode and cathode rotate in opposite directions create a swirling flow between the electrodes and bubbles spread out from the duct. This decreases internal resistance and increases conductivity and gives more effective electrolysis of water.

For vertical electrodes, the horizontal Lorentz force that is induced by a magnetic field parallel to the electrode has the greatest effect on the movement of the bubbles and significantly increases the conductivity. If vertical electrodes face vertical magnets, bubbles rotate vertically and do not detach from the surface of the electrodes significantly. For vertical electrodes with an upward exit, neither a perpendicular nor the parallel magnetic field causes movement of the bubbles.

These experimental results demonstrate the optimal layout for electrodes and a magnetic field that increases conductivity and the effectiveness of water electrolysis. The findings of this study can be used in a variety of fields including energy conversion, biotechnology, and an MHD thruster used in seawater.

## Supplementary Information

Supplementary Video Legends.

Supplementary Video 1.

Supplementary Video 2.

Supplementary Video 3.

Supplementary Video 4.

Supplementary Video 5.

Supplementary Video 6.

Supplementary Video 7.

## Data Availability

The datasets generated during the current study are available from the corresponding author on reasonable request. Movies for the experimental results demonstrated in the article are available as Supplementary Information.
