# Supplementary material for: The effect of magnetic field on the dynamics of gas bubbles in water electrolysis
Source: Sci Rep. 2021 Apr 30;11:9346. doi: 10.1038/s41598-021-87947-9 (PMC8087803; doi:10.1038/s41598-021-87947-9)
Supplement: Supplementary file 1 — Supplementary Video Legends. [file 41598_2021_87947_MOESM1_ESM.docx]

Supplementary Video Legends

Supplementary Video 1: Oxygen and hydrogen revolve in opposite direction

Supplementary Video 2: Oxygen bubbles revolve at lower anode facing N-pole (5 times slow down)

Supplementary Video 3: Hydrogen bubbles revolve at lower cathode facing N-pole

Supplementary Video 4: Oxygen and hydrogen bubbles respectively detached from the lower and upper platinum electrode (5 times slow down)

Supplementary Video 5: Oxygen and hydrogen bubbles respectively detached from the upper and lower platinum electrode (5 times slow down)

Supplementary Video 6: Oxygen and hydrogen bubbles respectively detached from the right and left platinum electrodes

Supplementary Video 7: Oxygen bubbles detached from the left platinum electrode and respectively repelled and attracted by the N-pole and S-pole of the magnet (5 times slow down)
